# Biomarkers for Precision Urothelial Carcinoma Diagnosis: Current Approaches and the Application of Single-Cell Technologies

**DOI:** 10.3390/cancers13020260

**Published:** 2021-01-12

**Authors:** Michelle Hong, George He, Siting Goh, Alvin Wei Xiang Low, Kae Jack Tay, Tony Kiat Hon Lim, Joe Yeong, Li Yan Khor, Tong Seng Lim

**Affiliations:** 1 A. Menarini Biomarkers Singapore Pte Ltd., Singapore 117440, Singapore; michelle.hong@mbiomarkers.com; 2 Department of Pathology, Singapore General Hospital, Singapore 169856, Singapore; georgehsy@gmail.com (G.H.); siting.goh@mohh.com.sg (S.G.); lim.kiat.hon@singhealth.com.sg (T.K.H.L.); 3 Department of Urology, Singapore General Hospital, Singapore 169854, Singapore; alvin.low@mohh.com.sg (A.W.X.L.); tay.kae.jack@singhealth.com.sg (K.J.T.); 4 Institute of Molecular and Cell Biology (IMCB), Agency for Science, Technology and Research (A*STAR), Singapore 138673, Singapore

**Keywords:** single cell, diagnostics, urothelial carcinoma, biomarker, non-invasive, cytology, cystoscopy, circulating tumor cells

## Abstract

**Simple Summary:**

Urothelial carcinoma (UC) is the most frequently diagnosed cancer of the urinary tract and is ranked the sixth most diagnosed cancer in men worldwide. About 70–75% of newly diagnosed UCs are non-invasive or low grade. Different tests such as urine cytology and cystoscopy are used to detect UC. If abnormal tissue is found during cystoscopy, then a biopsy will be performed. Cytology has low sensitivity for low-grade cancer while cystoscopy is invasive and costly. Detecting UC early improves the chances of treatment success. Therefore, many researchers have painstakingly identified urine biological markers for non-invasive UC diagnosis. In this review, we summarize some of the latest and most promising biological markers (including FDA-approved and investigational markers). We also discuss some new technologies that can aid research efforts in biological marker discovery for early UC detection.

**Abstract:**

Urothelial carcinoma (UC) is the most frequent malignancy of the urinary system and is ranked the sixth most diagnosed cancer in men worldwide. Around 70–75% of newly diagnosed UC manifests as the non-muscle invasive bladder cancer (NMIBC) subtype, which can be treated by a transurethral resection of the tumor. However, patients require life-long monitoring due to its high rate of recurrence. The current gold standard for UC diagnosis, prognosis, and disease surveillance relies on a combination of cytology and cystoscopy, which is invasive, costly, and associated with comorbidities. Hence, there is considerable interest in the development of highly specific and sensitive urinary biomarkers for the non-invasive early detection of UC. In this review, we assess the performance of current diagnostic assays for UC and highlight some of the most promising biomarkers investigated to date. We also highlight some of the recent advances in single-cell technologies that may offer a paradigm shift in the field of UC biomarker discovery and precision diagnostics.

## 1. Introduction

Bladder cancer (BC) is among the top 10 most common types of cancer worldwide, with around 550,000 new cases annually [1], and confers the highest financial burden to developed countries. BC accounts for around 3% of all new cancer diagnoses and 2.1% of all cancer-associated deaths [2], ranking it 6th highest in men and 17th in women worldwide in terms of absolute incidence. Among the 550,000 new BCs diagnosed globally in 2018, around 425,000 (77%) occurred in men and over 125,000 cases (23%) occurred in women [3].

The World Health Organization’s 2016 classification states that the three most common BCs are urothelial carcinoma (UC), squamous cell carcinoma, and adenocarcinoma. Among these, UC is the most common form, accounting for 90–95% of BC cases. BC is pathologically staged as non-invasive, stromal invasive, and muscle invasive.

Different tests and procedures are used to diagnose UC. The current diagnostic technologies include urinalysis, cystoscopy, urine cytology, biopsy, urine-based biomarkers, and clinical imaging such as computerized tomography (CT) urogram or retrograde pyelogram. Urine cytology is inexpensive and commonly used for initial detection of malignant cells, whereas cystoscopy with biopsy confirms the presence of the tumor [4] and allows for pathologic staging. While urine cytology is a useful tool for detecting high-grade UCs (up to 85% and 88% sensitivity and specificity, respectively), the sensitivity for the detection of low-grade UCs remains very low (10–43.6%). Cystoscopy has a low diagnostic accuracy, especially with flat urothelial carcinoma in situ (CIS), which is missed in up to 20% of all cases [4]. Cystoscopy can also be unreliable for distinguishing between benign reactive lesions and malignant lesions, particularly in cases of prior transurethral resection (TUR) or intravesical therapy [5].

New endoscopic technologies, such as fluorescence cystoscopy, narrow-band imaging, confocal laser endomicroscopy (CLE), and optical coherence tomography have been developed to improve the rate and accuracy of detection, although these techniques are invasive, expensive, and time-consuming [5]. Therefore, the development of novel non-invasive urinary tests to detect UC-specific biomarkers has increased over the last few decades [6,7,8].

**Figure 1:**
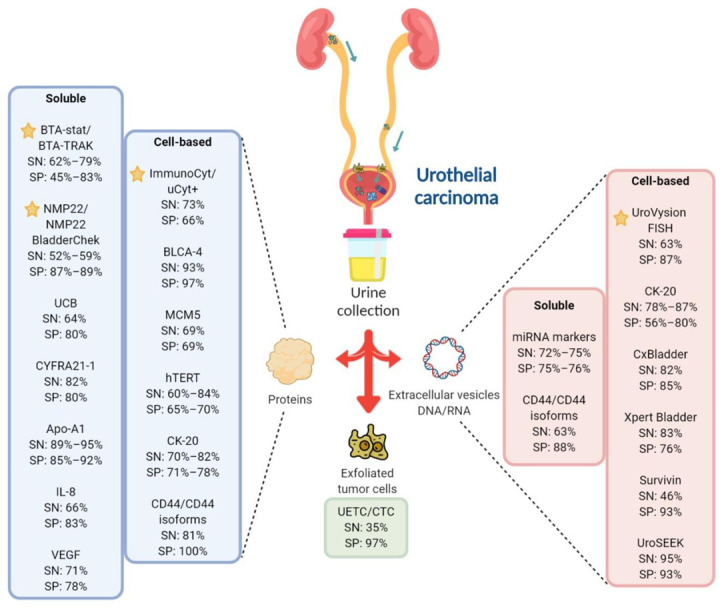
Overview of the FDA-approved and investigational urinary biomarkers for UC diagnosis. Most biomarkers, both soluble and cell-based, aims to detect either exfoliated tumor cells, protein or DNA/RNA changes in urine samples. Some of the promising investigational urinary biomarkers for UC and their sensitivity/specificity are shown and compared against the FDA-approved in-vitro diagnostic (IVD) tests. indicates FDA-approved IVD tests. 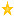 Created with Biorender.com.

The present review provides details of the current FDA-approved diagnostic assays for UC and examines some of the emerging and novel biomarkers (Figure 1, Table 1 and Table 2). While some studies are still in the preliminary stages, the purpose of this review is to highlight promising biomarkers with potential future diagnostic use in the clinic.

## 2. Current Research Gaps in UC Diagnosis

Around 70–75% of newly diagnosed UC cases are non-muscle invasive bladder cancer (NMIBC). The current gold standard NMIBC treatment is surgical removal via TUR. Due to the high rate (~70%) of recurrence after TUR, patients require an intensive follow-up regime that lasts many years following the initial diagnosis. This lifelong requirement for disease surveillance means UC is associated with the highest cost from diagnosis to death [48].

Urine cytology has high sensitivity in detecting high grade urothelial tumors (84%), but low sensitivity in low-grade tumors (16%) [49]; hence, urine cytology can miss low-grade NMIBC tumors. While cystoscopy remains the gold standard evaluation modality in the diagnosis of UC, it is invasive, costly, and the procedure is uncomfortable for the patient. Urine biomarkers offer a non-invasive approach to detect UC, especially for high-grade lesions and CIS, with higher sensitivity but lower specificity than urine cytology [50].

The Food and Drug Administration (FDA) has approved six urinary assays for clinical in-vitro diagnostic (IVD) use: BTA-stat, BTA-TRAK, NMP22, NMP22 BladderChek, ImmunoCyt/uCyt+, and UroVysion fluorescence in situ hybridization (FISH) (Table 1). While these FDA-approved IVD tests show high sensitivity in the detection of high-grade and late-stage UC, they are unable to detect low-grade malignancies and tend to give false positive results for benign inflammatory conditions. As such, they cannot be used as stand-alone diagnostic tests for UC and are used in conjunction with urine cytology or other diagnostic tests.

Novel and emerging biomarkers are continuously being developed and have shown higher sensitivity, specificity, and accuracy than urine cytology and current FDA-approved tests (Table 2). However, their use in clinical practice requires validation studies using independent cohorts and long-term follow-up. At present, there is no non-invasive biomarker that has been recommended to replace the gold standard methods currently used to detect UC.

## 3. FDA-Approved and Investigational UC Biomarkers

In the following sub-sections, we highlight the various FDA-approved IVD tests and some promising emerging/novel biomarkers for UC diagnosis.

### 3.1. FDA-Approved IVD Tests for UC Diagnosis

Increasing research efforts have been placed in identifying a suitable urinary biomarker to reduce the necessity of invasive cystoscopy. The various FDA-approved tests are a major step towards this direction. However, the inclusion of large proportions of high-grade tumors inflates the sensitivity and specificity of many FDA-approved tests, and thus the problem of identifying low grade tumors remains.

#### 3.1.1. Bladder Tumor Antigen (BTA) Assay

The BTA-TRAK and BTA-stat kits use sandwich immunoassay and the colorimetric antigen-antibody reaction, respectively, to detect human complement factor H-related protein in urine. These soluble factors are secreted by bladder tumor cells during stromal invasion and inhibit the complement cascade to prevent cell lysis, which may allow tumor cells to escape host immunity [51]. The BTA-TRAK kit is a quantitative test that requires processing in a suitably equipped laboratory, whereas the BTA-stat kit provides immediate qualitative results. The BTA-TRAK and BTA-stat kits have a sensitivity of around 65% and specificity of 74–77% [13,52]. A meta-analysis of 13 studies that reviewed 3175 patients found the sensitivity and specificity of the BTA-stat kit to be 64–69% and 73–77%, respectively [9]. However, the studies included in this meta-analysis mainly compared individuals with high-grade UC with control groups. Goodison et al. reported a sensitivity of 79% and specificity of 83% for the BTA-TRAK kit [8]. This was supported by a meta-analysis of five studies including individuals with either low or high-grade UC [10]. However, as complement factor H-related protein is also found in the blood and is not unique to UC, other causes of hematuria, such as post-treatment hematuria (e.g., intravesical chemotherapy or immunotherapy), infection, or recent instrumentation, can result in false positives that may significantly decrease the specificity of these kits. Despite this limitation, these tests have been approved by the FDA for the detection of UC in symptomatic patients or those under surveillance for UC.

#### 3.1.2. Nuclear Matrix Protein 22 (NMP22)

The NMP22 test kit uses a colorimetric antigen–antibody technique to detect NMP22 in urine. NMP22 is a biomarker that is derived from urothelial cell death and is elevated in the urine of UC patients [53]. Data from 19 studies and 23 systemic reviews suggest a sensitivity of 52–59% and specificity of 87–89% [11]. Similar to the BTA-TRAK and BTA-stat kits, the NMP22 test kit is also prone to false positive results as NMP22 is released during apoptosis, which also occurs during infection and inflammation, and is not specific to malignancy [54]. The quantitative NMP22 Bladder Cancer ELISA Test kit and the NMP22 BladderChek point-of-care (POC) test are FDA-approved for the detection and surveillance of UC.

#### 3.1.3. ImmunoCyt/uCyt+ Assay

The ImmunoCyt test is a triple immunofluorescent monoclonal antibody assay that measures the glycosylated form of carcinoembryonic antigen (CEA) and two mucins (LDQ10 and M344) that are specifically found on malignant exfoliated urothelial cells [55]. ImmunoCyt reportedly has a sensitivity of 74–87% and specificity of 62–78%, with a positive predictive value (PPV) of 26–67% and negative predictive value (NPV) of 91–96% [56,57]. In agreement with this, a meta-analysis by He et al. reported a sensitivity of 73% and specificity of 66% [12]. False positives due to infection and inflammation [e.g., following bacillus Calmette–Guérin (BCG) treatment] affect the specificity of the kit. He et al. reported that the ImmunoCyt test had a higher sensitivity, but lower specificity than the urine cytology test. The ImmunoCyt kit can be combined with urine cytology as a surveillance tool as it has ~80% sensitivity for low-grade tumors and almost 100% sensitivity for high-grade tumors [55].

#### 3.1.4. UroVysion FISH

The UroVysion FISH test detects genetic markers, specifically aneuploidy for chromosomes 3, 7, and 17, and loss of 9p21 locus. In a meta-analysis, the reported sensitivity and specificity were 63% and 87%, respectively [13]; however, it lacks sensitivity for low-grade UC. UroVysion FISH is FDA-approved and seems to be useful for predicting recurrence in the setting of a negative surveillance cystoscopy and “atypical” urine cytology [58] as well as other specific scenarios. Some examples include disease recurrence detection with an “atypical” cytology following intravesical BCG treatment of high-grade NMIBC and diagnosis of upper urinary tract UC in an “atypical” upper tract washing cytology. Pitfalls of UroVysion FISH include the complexity of the assay and the requirement for a skilled cytopathologist, a high false-positive rate, and a lack of consensus on the criteria to evaluate abnormal cells.

### 3.2. Novel/Investigational Biomarkers for UC Detection

Some novel candidate biomarkers have been identified that require further validation and, as such, are not yet FDA-approved. The sensitivity and specificity of some of these markers are better than urine cytology or current FDA-approved tests, especially for low-grade tumors. These investigational biomarkers are grouped into two categories: cell-based and soluble. We discuss the biomarkers belonging to these two categories in the sections below, as well as CD44/CD44 isoforms and microRNA (miRNA) markers that can be classified as either cell-based or soluble.

#### 3.2.1. Cell-Based Biomarkers

Cell-based biomarkers rely on the detection of urine exfoliated tumor cells (UETCs) or the proteins (surface or intracellular) or genomic content (DNA or RNA) of such cells. These include bladder cancer 4 (BLCA-4), minichromosome maintenance 5 (MCM5), human telomerase reverse transcriptase (hTERT), circulating tumor cells (CTCs), cytokeratin 20 (CK-20), CxBladder, Xpert Bladder, Survivin, UroSEEK, and AssureMDX. We discuss each of these cell-based urinary biomarkers and their diagnostic performance below.

##### BLCA-4

BLCA-4 is a nuclear transcription factor expressed in bladder tumors, especially during the early stages of the disease [14]. It is absent in healthy bladder tissue but has a high sensitivity in detecting low-grade UC. BLCA-4 can be measured using commercially available ELISAs. A meta-analysis of BLCA-4 in UC diagnosis involving 1119 individuals indicated a high pooled sensitivity (93%) and specificity (97%) [14]. However, larger prospective studies are required to translate this biomarker into clinical use.

##### MCM5

The MCM family of proteins assemble into hexameric complexes with DNA helicase activity and are vital for the initiation of DNA synthesis [59]. MCM proteins are dysregulated and overexpressed in hyperproliferative and malignant cells [60]. Increased MCM5 levels in the urine samples of patients, measured by immunofluorometric assay, is predictive of UC [61]. Kelly et al. demonstrated that MCM5 levels could be used to discriminate between patients with and without UC, with 69% sensitivity and specificity [15]. The MCM5 test has similar specificity but significantly higher sensitivity than cytology [15], and can detect all grades and stages of UC.

##### hTERT

Telomerase is a ribonucleoprotein that synthesizes telomeres at the ends of chromosomes, thus ensuring genomic stability [62]. Several tumors, including UC, are characterized by telomerase hyperactivity that protects the chromosomes of cancer cell and prevents them from dying. In a retrospective analysis of 101 cell blocks from UC patients, Khalbuss and Goodison reported that hTERT showed a higher diagnostic sensitivity (84.8%) but lower specificity (65.2%) than cytology (~65% and ~95%, respectively) [16]. Allison et al. evaluated the performance of hTERT in 500 urinary tract cytology specimens and reported a sensitivity of 60.6% and specificity of 70.4% [17]. Overall, hTERT could be used as an adjunct to urine cytology to aid the identification of patients with an increased risk of high-grade UC.

##### CTCs/UETCs

CTCs or urine exfoliated tumor cells (UETCs) are malignant epithelial cells that are shed from the primary tumor into bodily fluids (e.g., urine) and can be indicative of micrometastatic disease [63]. CTCs may harbor important information about the primary tumor that could have important prognostic and diagnostic value. CTCs are detected in clinical settings using immunocytochemistry, reverse-transcription polymerase chain reaction (RT-PCR), flow cytometry, or the CellSearch system, which is the only FDA-approved CTC test. It is well established that the presence of CTCs is an indicator of poor prognosis in breast, colorectal, and gastric cancers [64,65,66,67]. A meta-analysis of 30 published studies involving 2161 UC patients showed that CTC positivity was significantly associated with tumor stage, histological grade, metastasis, and lymph node metastasis [68]. It was also significantly associated with poor overall survival, progression/disease-free survival, and cancer-specific survival. As CTC detection in UC has relatively low sensitivity (35%) but high overall specificity (97%), it is not currently used as a screening/diagnostic test; however, it is used as a method to confirm UC diagnosis [68,69]. The ability to conjugate different antibodies to Ferrofluid in the CellSearch system may allow for the identification of new CTC biomarkers for more sensitive detection. Further well-designed, large-scale prospective studies are required to determine the potential use of CTCs as a biomarker for UC diagnosis.

##### CK-20

Cytokeratins belong to a family of >20 intermediate filament proteins that are expressed in epithelial cells. CK-20 is expressed in UC but not normal urothelial cells and is measured non-invasively in urine via RT-PCR or immunostaining. It has also been reported as a biomarker for UC detection. A meta-analysis of 27 diagnostic studies reviewing 3473 participants concluded that the urine CK-20 test had a pooled sensitivity of 78–87% and specificity of 56–80% [22], but showed poor performance for low-grade disease. Other reports, however, showed that CK-20 positive atypical urothelial cells were indicators of low-grade UC [70,71,72]. CK-20 immunostaining for UC detection showed good sensitivity (70–82%) and specificity (71–78%), indicating the potential use of CK-20 as a biomarker for UC detection [19,20,21].

##### CxBladder

CxBladder is a clinically validated multiplex mRNA test used to measure the urine concentration of five RNA markers (CDC2, HOXA13, MDK, IGFBP5, and CXCR2) in UC patients [73]. CXCR2 is expressed by neutrophils and its levels are increased in non-malignant inflammation that increases the cellularity of voided urine samples [23]. The measurement of CXCR2 levels is hypothesized to reduce the chance of false-positive results in patients with acute or chronic urothelial inflammation. The expression levels of these five RNA markers are determined by quantitative RT-PCR (RT-qPCR). O’Sullivan et al. reported that CxBladder has a sensitivity of 82% and specificity of 85% [23]. CxBladder can detect high-grade tumors with a higher accuracy than current FDA-approved NMP22 tests and cytology. Importantly, Cxbladder can distinguish between low-grade Ta stage tumors and other UCs with a sensitivity of 91% and specificity of 90%, respectively.

##### Xpert Bladder

Xpert Bladder Cancer Monitor (Xpert) measures the expression of five mRNA targets (ABL1, CRH, IGF2, UPK1B, and ANXA10) that are often overexpressed in NMIBC and are detected in voided urine samples [24]. The assay is performed in a self-contained cartridge using the GeneXpert System, which automates and integrates cell lysis, nucleic acid amplification, and target sequence detection using RT-qPCR. It is a fast, easy-to-use, and robust assay. A study of 239 patients by Valenberg et al. found that the Xpert assay had a sensitivity of 76% and specificity and 85% [24]. The Xpert assay has a higher sensitivity and NPV than cytology and UroVysion FISH; however, the specificity seems to be only marginally higher than UroVysion FISH and lower than cytology. The Xpert assay has high NPVs of 93% and 98% and thus might make an important contribution to monitoring patients with NMIBC. A high NPV suggests that urologists can consistently exclude diseases with negative results and reduce the number of invasive cystoscopies for intermediate-to-high-risk NMIBC, thus reducing costs and patient discomfort [4].

##### Survivin

Survivin is an anti-apoptotic protein that is almost exclusively expressed in the malignant epithelium [74]. Survivin and NMP22 levels were measured by Shariat et al. in voided urine samples from 117 UC patients undergoing cystoscopy and 92 control individuals [25]. They found that survivin displayed higher sensitivity (64%), specificity (93%), PPV (92%), and NPV (67%) than NMP22 or urine cytology. Higher levels of survivin were also associated with more advanced histologic grades [75]. As this is the only published study to-date to evaluate the role of urinary survivin in the follow-up of NMIBC, this assay remains experimental and requires further development and validation.

##### UroSEEK

UroSEEK is a novel, non-invasive urine-based biomarker that is measured by applying massively parallel sequencing to cellular DNA to detect UC mutations involving the TERT gene promoter and 10 other genes (FGFR3, TP53, CDKN2A, ERBB2, HRAS, KRAS, PIK3CA, MET, VHL, and MLL) combined with aneuploidy assessment [76]. These genes are involved in signaling pathways associated with NMIBC. Springer et al. reported that UroSEEK displayed high sensitivity and specificity (95% and 93%, respectively) and had a higher performance than urine cytology in low-grade tumors [26]. In an early detection setting, UroSEEK demonstrated high sensitivity and specificity (96% and 88%, respectively), and had a significant lead time to clinical cancer diagnosis, as 7 out of 22 patients from an early detection cohort were detected 6 months prior to clinical diagnosis [76]. UroSEEK shows promising potential in diagnosing patients with atypical cytology.

##### AssureMDX

AssureMDX is a laboratory-developed test that identifies DNA mutations in three genes (FGFR3, TERT, and HRAS) and methylation in another three genes (OTX1, ONECUT2, and TWIST1) in urine samples [77]. In a large multicenter prospective study of 977 patients with primary NMIBC, the sensitivity for recurrent detection was 57% for low-grade tumors and 83% for high-grade tumors [28]. In another multicenter study involving 97 UC patients, AssureMDX demonstrated a sensitivity of 93% and specificity of 86% for UC diagnosis [27]. The AssureMDX assay thus shows potential as a diagnostic tool in patients with low-grade tumors to identify high-grade tumors earlier.

#### 3.2.2. Soluble Biomarkers

UC cells can release soluble factors into the urine in the form of proteins or cell-free DNA/RNA contained in extracellular vesicles. A number of promising soluble biomarkers have been investigated, including urinary bladder carcinoma antigen (UBC), CYFRA21-1, apolipoprotein-A1 (Apo-A1), interleukin-8 (IL-8), vascular endothelial growth factor (VEGF), C–C motif chemokine 18 (CCL18), hyaluronic acid (HA) or hyaluronidase (HAse) and soluble Fas (sFas). We discuss each of these in details below.

##### UBC Test

The UBC test measures soluble fragments of cytokeratins 8 and 18 in urine; these factors have a role in tumor invasion [78]. The UBC test is available in two different formats: a quantitative ELISA-based assay (UBC IRMA) and a qualitative POC-based assay (UBC Rapid). The mean sensitivity and specificity reported in 11 studies involving 753 patients and 1072 controls were 64.4% and 80.3%, respectively [7]. The overall sensitivity increased to 77.4% when used in combination with cytology [79]. Furthermore, high CK8 and CK18 levels detected in urine by the UBC Rapid test enable the distinction between high- and low-grade UC [29].

##### CYFRA 21-1

CYFRA 21-1 detects soluble cytokeratin 19 fragments by ELISA or immunoradiometric assay. Several studies have reported the ability of CYFRA 21-1 to distinguish between UC patients from controls, achieving sensitivity of 70–90% and specificity of 73–86% [7,30,80]. Huang et al. conducted a meta-analysis of 16 studies, with a total of 1252 patients and 1233 controls [30]. The mean reported sensitivity and specificity of urinary CYFRA21-1 were 82% and 80%, respectively. This assay has high sensitivity for the detection of high-grade and CIS tumors, but not for early detection.

##### Apo-A1

Apo-A1 is a major high-density lipoprotein that is highly expressed and secreted in UC patients [33,81]. In a study conducted on 223 patients with UC and 153 without UC, soluble Apo-1 urinary concentrations measured using commercial ELISA were significantly higher in UC patients compared with healthy individuals, with a sensitivity of 89% and specificity of 85% [31]. Another study by Chen et al. including 86 patients and 62 controls reported an even higher sensitivity (95%) and specificity (92%) [33]. In an early detection setting, Apo-A1 concentrations were shown to be increased in the urine of patients with low-grade transitional cell carcinoma (TCC) and increased further in aggressive UC [32]. The diagnostic sensitivity and specificity were 91.6% and 85.7%, respectively. Apolipoproteins are moderately abundant in plasma and hence urinary concentrations could be influenced by hematuria. However, the high sensitivity and specificity of the Apo-A1 test, combined with its potential for early diagnosis highlights Apo-A1 as a promising diagnostic urinary biomarker for UC.

##### IL-8

Human IL-8 is a chemokine produced by bladder epithelial cells and is elevated in UC [36,82,83]. IL-8 levels in the urine of UC patients have shown a weighted sensitivity and specificity of 66.4% and 83.1%, respectively, from the combined data of four studies [34,35,36,37]. Urinary IL-8 concentrations are elevated in patients with TCC compared with healthy controls, and increase with increasing stage but not grade, indicating a correlation between IL-8 production and tumor invasiveness or angiogenesis [36]. IL-8 has also been reported in other multianalyte panels, where it showed high sensitivity and specificity in detecting UC [8].

##### VEGF

VEGF is involved in tumor angiogenesis and is secreted into the urine by UC cells [84]. VEGF levels are measured by ELISA: elevated VEGF levels are associated with a higher disease recurrence risk in patients with previous NMIBC [85]. The weighted sensitivity and specificity reported in six different studies based on 509 patients and 389 controls were 71.4% and 78.1%, respectively [8,34,35,38,39,40]. Similar to IL-8, VEGF has also been reported in several multianalyte panels for UC detection with high sensitivity and specificity [8].

##### CCL18

CCL18 is a soluble cytokine involved in immunoregulatory and inflammatory processes [41]. It promotes cancer cell invasiveness by triggering integrin clustering and enhancing adherence to the extracellular matrix [41]. CCL18 is measured using commercially available ELISAs. Urinary CCL18 is undetectable in most individuals without UC but is significantly expressed in patients with UC. CCL18 can differentiate between patients with UC and healthy individuals with a high sensitivity (88%) and specificity (86%) [41]. Most studies on CCL18 have focused on high-grade and high-stage disease, while studies in patients with low-grade and low-stage disease are limited.

##### HA/HAse

HA is a glycosaminoglycan that is involved in cell adhesion and proliferation and promotes tumor growth and metastasis. HAse is a glycosidase that mainly degrades HA into small fragments that promote tumor angiogenesis [86]. HA and HAse have been found to be elevated in the urine of UC patients measured using ELISA and RT-PCR/substrate (HA)-gel zymography, respectively. HA and HAse, both individually and combined, showed high potential diagnostic value as non-invasive urinary biomarkers [87,88]. A meta-analysis of five different studies including a total of 981 participants demonstrated that the HA–HAse test showed superior sensitivity (90.8%) and specificity (82.5%) than HA and HAse alone, indicating that the combined HA and HAse measurement has potential as a biomarker for UC detection [42]. Further validation in larger, multicenter trials is required to translate this test into clinical practice.

##### sFAS

Urinary sFas is a cleaved cell-surface receptor that belongs to the tumor necrosis factor protein family commonly expressed in UC [89]. The dysregulation of Fas-mediated apoptosis is hypothesized to lead to UC progression and development [89]. Serum sFas levels are reportedly three times higher in UC patients than healthy individuals. In a study performed by Srivastava et al., the measurement of urinary sFas concentrations showed high sensitivity (88.0%) and specificity (89.1%) in the diagnosis of TCC of urinary bladder for both primary and recurrent disease [43]. The measurement of urinary sFas also showed greater sensitivity in detecting low-grade UC compared with cytology (88.01% vs. 66.67%, respectively).

#### 3.2.3. Cell-Based/Soluble Biomarker

As mentioned, there are two potential biomarkers that can be classified as either cell-based or soluble. These biomarkers can be expressed by UETCs or they can be secreted extracellularly by proteolytic cleavage of the membranous form/alternative splicing (e.g., CD44/CD44 isoforms) or encapsulated into extracellular vesicles (e.g., miRNA markers).

##### CD44/CD44 Isoform

CD44 is cell surface adhesion protein that has important roles in various biological processes, including lymphocyte homing and activation, cell motility, and cell–matrix interactions [90]. Changes in CD44 expression levels are commonly associated with tumor invasion and metastasis [91]. A study involving 136 patients, including 111 histologically diagnosed with UC, showed an overall sensitivity and specificity of 63.1% and 88.9%, respectively, for urinary CD44 [46]. Urinary CD44 was measured using RT-qPCR and was positively associated with tumor aggressiveness in UC. Woodman et al. showed that CD44 isoforms in protein lysates of exfoliated tumor cells in the urine (detected by ELISA) could be reliably detected in UC, with 81.1% sensitivity and 100% specificity [47]. The presence of hematuria can interfere with this assay but does not limit the clinical potential of this biomarker. Repeated testing and further validation are required to determine the potential use of CD44 as a promising biomarker for reliable, routine, and noninvasive detection of early UC.

##### miRNA Markers

miRNAs are short (21–23 nucleotides in length) non-coding RNAs that regulate gene expression at the post-transcriptional level. The diagnostic value of miRNAs in patients with UC has been reported in numerous studies (reviewed in [92]). Urinary miRNAs can be measured by RT-PCR in urine supernatants or sediments [44]. Recently, high-throughput miRNA profiling has been performed using next-generation sequencing (NGS) [93]. A multi-miRNA assay was reported to have higher diagnostic sensitivity than a single miRNA assay [94]. A meta-analysis of 23 studies, including 719 patients and 494 controls, was conducted to evaluate the diagnostic potential of miRNAs in UC, and showed that urinary miRNAs have a pooled sensitivity of 75% and specificity of 75% in diagnosing UC [44]. Another meta-analysis of 31 studies, including 1556 patients and 1347 controls, reported a sensitivity and specificity of 72% and 76%, respectively [45]. miRNA assays may serve as potential diagnostic tools for UC; their clinical application now requires further validation in large prospective studies.

## 4. Single-Cell Technologies

**Figure 2:**
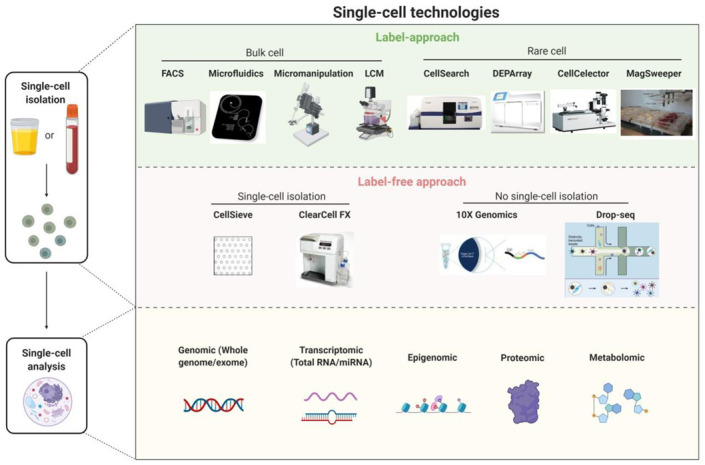
An overview of single-cell technologies. Single-cell technologies involve two steps: single-cell isolation followed by single-cell analysis. Single-cell isolation includes label approaches (e.g., FACS, microfluidics, micromanipulation, LCM, CellSearch, DEPArray, CellCelector, and MagSweeper) and label-free approaches (e.g., CellSieve and ClearCell FX). Some single-cell technologies such as 10× genomics and drop-seq do not involve single-cell isolation before single-cell analysis. Single-cell analysis techniques include genomics (whole genome/exome), transcriptomics (total RNA/miRNA), epigenomics, proteomics and metabolomics. FACS, flow assisted cell sorting, LCM, laser capture microdissection. Created with BioRender.com.

Single-cell technology has made encouraging progress in recent years such that it now provides the means to detect, isolate, and analyze rare target cells such as CTCs, cancer stem cells, or pathogenic immune cells to ultimately guide individualized treatment strategies. Most single-cell technologies involve two steps: single-cell isolation and analysis (Figure 2).

Single-cell isolation is the upstream process required prior to single-cell analysis and includes: (1) marker/phenotype-based methods for isolating single cells from bulk cell populations (e.g., fluorescence-activated cell sorting, microfluidics, micromanipulation, and laser-capture microdissection) or from rare cell populations e.g., CellSearch (Menarini Silicon Biosystems), DEPArray (Menarini Silicon Biosystems), CellCelector (Automated Lab Solutions) and MagSweeper (Illumina Inc.), and (2) label-free approaches based on the biophysical properties of cells such as size, shape, density, and stiffness [e.g., nanofabricated filters (CellSieve) and ClearCell FX (Clearbridge BioMedics)].

Some single-cell technologies (e.g., 10× Genomics, Drop-Seq) do not isolate individual target cells prior to single-cell analysis. Instead, these technologies involve encapsulating single cells with single barcoded beads in nanoliter-sized droplets and allow ultra-high-throughput phenotyping and molecular characterization of all individually encapsulated cells in the samples. Cell identity is then inferred through reverse bioinformatics analysis of the high throughput data (e.g., transcriptome analysis).

Single-cell analysis reveals the heterogeneities in morphology, function, composition, and genetic make-up of apparently identical cells. Recent advances in single-cell analysis can overcome the difficulties arising in the diagnostics for a targeted model of disease due to cell heterogeneity. Single-cell analysis techniques include genomic (whole genome/whole exome), transcriptomic, epigenomic, proteomic, and metabolomic profiles of cancer cells. Among these, single-cell genomic analysis has shown the most encouraging progress [18,95,96].

### Applications of Single-Cell Technologies for UC Diagnosis

In UC, tumor cells that carry key genetic information of the primary tumor are shed directly by the growing tumor of the bladder into bodily fluids, such as blood or urine, making them a promising liquid biomarker for UC detection. However, in UC patients, the detection of UETCs is complicated by the presence of other urinary components such as normal urothelial cells, red blood cells, crystals, urinary cylinders, and other impurities. Furthermore, the extreme rarity and heterogeneity of CTCs/UETCs makes detecting these cells a challenging endeavor in UC diagnosis.

Advancements in single-cell technologies have increased the sensitivity and reliability of the methodologies for CTC detection, such as the CellSearch system and microfluidic-based techniques. CellSearch is an FDA-approved CTC test for the analysis of blood samples from patients with metastatic breast, prostate and colorectal cancer [97,98,99,100]. The CellSearch system has been successfully used to detect CTCs derived from patients with UC in multiple studies [68,101]. Although the number of patients enrolled and the sensitivity remains low, these early data provide a proof-of-concept for the identification of UETCs in UC patients using the CellSearch system. Microfluidic-based techniques to detect cancer cells in urine have also been recently developed and provide a novel approach to UC diagnosis. Several microfluidic devices that can detect rare UETCs have been developed [102,103], including a single-cell microscopic observation device for detecting tumor cells using droplet microfluidic technology [104].

Single-cell analysis can have broad applications in the UETC diagnostic workflow. Single-cell sequencing (SCS) such as whole-genome sequencing (WGS) was used by Chen et al. to analyze copy number aberrations (CNAs) in 12 UETCs captured using a novel microfluidic immunoassay approach [102]. The identities of the captured UETCs were successfully verified based on their genomic instability profiles, demonstrating the potential diagnostic capability of these methods for patients with UC. Single-cell miRNA profiling and single-cell epigenomic profiling also provide the exciting possibility of linking genetic and transcriptional heterogeneity in the context of cancer biology, leading to improved cancer diagnosis. The relative stability of miRNAs and DNA methylation has led to the development of diagnostic urinary biomarkers in UC [105,106,107,108,109,110,111]. Single-cell miRNA and epigenomic profiling have the potential to identify modifications specific to UETCs and thus guide the appropriate diagnosis. Finally, single-cell metabolomics analyses can detail the metabolic characteristics of UETCs at the single-cell level, thus identifying potential biomarkers that could be used for early UC diagnosis.

## 5. Discussion and Future Perspectives

The high recurrence rate and requirement for invasive diagnostic and monitoring methods, such as cystoscopy, makes UC the most expensive human cancer from diagnosis to death. While cystoscopy and urine cytology remain the gold standard for initial UC diagnosis and staging, there is a push to discover novel, non-invasive biomarkers for the diagnosis, prognosis, and follow-up of UC using liquid biopsy samples. As such, the past decade has seen the development of different diagnostic and monitoring systems for UC patients, based on gene expression or protein biomarkers in urine samples.

The degradation of cells, proteins, DNA, and RNA in urine samples depends on time and temperature, resulting in a different quantity and quality of cells and soluble factors, and thus highly variable sensitivities and specificities of such biomarkers in the urine. However, urinary biomarkers are attractive because the testing is non-invasive and cost-efficient, and the sample collection is easy compared to the invasive cystoscopy gold standard tests. 

Urinary biomarkers, which can be either cellular or soluble, have their inherent advantages and disadvantages. Soluble biomarkers are easy to collect and the tests are usually carried out using simple laboratory techniques that are quick and relatively inexpensive. However, the main drawback of such techniques is low sensitivity. Furthermore, the original cellular source of these soluble biomarkers might not be obvious, and their stability varies depending on the types of biomarkers. On the other hand, cellular biomarkers are specific and allow single-cell analysis. Cellular biomarkers can be used for initial enrichment and/or isolation for further downstream molecular/metabolomic analyses. Such cellular biomarkers can also be used in combination with other visualization techniques (e.g., cystoscopy, IHC) to confirm the diagnosis. The major limitation of cellular biomarkers is the rarity of UETCs. Other drawbacks include the complexity of the techniques that often require highly skilled and trained personnel and specialized laboratories, and the associated high cost.

The current FDA-approved urinary biomarkers for the diagnosing and monitoring UC are unable to replace urine cytology due to their low sensitivity and specificity, particularly in detecting low-grade tumors (e.g., NMP22 test). Other tests have high costs that limit their use in daily clinical practice (e.g., UroVysion FISH). Several novel biomarkers have been identified that have the potential to detect NMIBC with high accuracy and NPV, outperforming conventional urine cytology methods in detecting low-grade tumors. In our opinion, urinary markers that have been trialed and tested in low grade NMIBC settings with sufficient sensitivity and specificity, such as hTERT, CK-20, CxBladder, Survivin, UroSEEK, AssureMDX, UBC, HA/HAse, and sFAS have great potential and could serve as powerful tools if they could be further developed, refined, and validated with large, randomized, and prospective cohort trials.

UC diagnosis can now leverage on single-cell technologies to detect, isolate, and analyze rare cancer cells such as CTCs and cancer stem cells or even pathogenic immune cells to ultimately guide individualized treatment strategies. Although emerging biomarkers are being continuously developed, especially for low-grade disease, there is still a significant lack of external validation using independent, large-scale cohorts with long-term follow up.

It is important to note that potential urinary biomarkers should complement, rather than substitute, conventional urine cytology and ultimately replace cystoscopy. This point is especially important for low-grade UC, given the fundamental weakness of cytology in the diagnosis or prognosis of low-grade UC. A complementary approach should also include cellular-based markers, rather than soluble markers, to allow for a direct comparison of such biomarkers with morphological characteristics of urothelial cells.

## 6. Conclusions

UC is a challenging disease in terms of its diagnosis and surveillance. Urinary biomarkers have the potential to improve current diagnostic strategies, but they do not have sufficient sensitivity to safely replace cystoscopy in this setting. Future studies should focus on the identification and validation of biomarkers for early detection, particularly for low-grade and low-stage NMIBC. Diverse and rapidly evolving single-cell technologies provide remarkable opportunities for cancer biomarker discovery, and it is possible that in the next few years, a generation of new biomarkers using molecular personalized medicine approaches will enter the clinical setting. Moving forward, the development of highly sensitive and specific urinary assays coupled with single-cell technologies might revolutionize UC diagnosis, allowing for improved, early detection of the disease, ultimately improving UC treatment.

## Figures and Tables

**Table 1:** FDA-approved urinary biomarkers for urothelial carcinoma (UC) in-vitro diagnosis.

Test	Biomarker	Type	Sample Material	Method	SN (%)	SP (%)	P (n)	C (n)	Remark	Reference
BTA-stat	Human complement factor H-related protein	Soluble	Protein	Dipstick immunoassay or POC test	64–69 *	73–77 *	3175	–	High false positive rates	[9] (meta- analysis)
BTA-Trak		Soluble	Protein	ELISA	79	83	64	63		[8]
					62–71 *	45–81 *	829	–		[10] (meta- analysis)
NMP22/ NMP22 BladderChek	NMP22	Soluble	Protein	ELISA or POC test	52–59 *	87–89 *	5291	–	Better at detecting high-grade UC; false positives in hematuria or inflammatory bladder conditions	[11] (meta- analysis)
Immuno-cyt/ uCyt+	High-MW form of glycosylated CEA and mucin-like antigen	Cellular	Protein	Immunocyto chemistry	73 *	66 *	1602	–	Unaffected by hematuria or inflammatory conditions; superior sensitivity to detect early pathological stage than cytology; test results highly dependent on specimen stability and handling	[12] (meta- analysis)
UroVysion FISH	Aneuploidy for chromosomes 3, 7, and 17, and loss of 9p21 locus	Cellular	DNA	FISH	63 *	87 *	3445	–	Complex assay that requires skilled cytopathologist; low sensitivity in the detection of low-grade UC; high rate of false positives; lack of consensus on the criteria to evaluate abnormal cells	[13] (meta- analysis)

UC, urothelial carcinoma; C, control; CEA, carcinoembryonic antigen; MW, molecular weight; NMP22, nuclear mitotic apparatus protein; P, patient; POC, point-of-care; SN, sensitivity; SP, specificity; FISH, fluorescence in situ hybridization. * weighted or pooled sensitivity/specificity.

**Table 2:** Novel/investigational urinary biomarkers for UC diagnosis.

Biomarker/ Test	Description	Type	Sample Material	Method	SN (%)	SP (%)	P (n)	C (n)	Remarks	References
BLCA-4	Nuclear transcription factor	Cellular	Protein	ELISA	93 *	97 *	1119 (total participants)		High sensitivity and specificity for UC detection; further validation required	[14] (meta- analysis)
MCM5 ^	MCM family of proteins that assemble into hexameric complexes with DNA helicase activity; vital for DNA synthesis	Cellular	Protein	Immuno- fluorometric assay	69	69	210	1354	Mix of low and high-grade patients; higher sensitivity but similar specificity compared with cytology	[15]
hTERT ^	Catalytic subunit of telomerase, a ribonucleoprotein that synthesizes telomeres at the ends of chromosomes, thus ensuring genomic stability	Cellular	Protein	Immunocytochemistry	84.8	65.2	101	–	Higher sensitivity than cytology, regardless of tumor grade and stage; lower specificity than cytology; may be used as an adjunct to cytology to identify patients with increased risk of high-grade UC	[16]
					60.6	70.4	500	–		[17]
CTCs ^	Malignant epithelial cells that are shed from the primary tumor into bodily fluids	Cellular	Protein	Immuno magnetic enrichment (CellSearch)	35 *	97 *	2161	–	The only FDA-approved CTC test; Possibility of staining with different antibodies which allows for the identification of new CTC biomarkers	[18] (meta- analysis)
CK-20	Cytokeratins are components of cytoplasmic intermediate filaments found in epithelial cells; CK-20 is expressed in urothelial carcinoma but not normal urothelial cells	Cellular	Protein	Immuno- staining	70	71	42	17	Higher sensitivity than urine cytology as a UC screening test, especially for low-grade low-stage tumor	[19]
					80	78	50	20		[20]
					82	77	174	–		[21]
			RNA (mRNA)	RT-PCR	78–87	56–80	3473	–	Poor performance for low-grade tumors	[22] (pooled analysis)
CxBladder	mRNA expression of genes (IGF, HOXA, MDK, CDC, and IL8R)	Cellular	RNA (mRNA)	RT-qPCR	82	85	66	419	Can distinguish between low-grade Ta tumors and other detected UC with high sensitivity and specificity	[23]
Xpert Bladder ^	mRNA expression of genes (CRH, IGF2, UPK1B, ANXA10, and ABL1)	Cellular	RNA (mRNA)	RT-qPCR	83	76	239	508	Mainly high-grade patients	[24]
Survivin	Inhibitor of apoptosis gene	Cellular	DNA	Bio-dot test	64	93	117	92	High sensitivity for detecting low-stage and low-grade UC; more accurate than cytology and NMP22 test; requires further validation	[25]
UroSEEK	Mutations in FGFR3, TP53, ERBB2, CDKN2A, KRAS, HRAS, MET, PIK3CA, MLL, and VHL and TERTp alterations	Cellular	DNA	Massively parallel sequencing- based assay (NGS/ Sanger sequencing)	95	93	570	–	Higher performance than urine cytology in low-grade tumors	[26]
AssureMDX	Mutation analysis in FGFR3, TERT, and HRAS genes and methylation analysis in OTX1, ONECUT2, and TWIST1 genes	Cellular	DNA	PCR	93	86	97	103	Mix of high and low-grade patients tested	[27]
					57–83	59	977	–	Patients with primary NMIBC	[28]
UBC ^	Soluble fragments of cytoskeletal proteins 8 and 18	Soluble	Protein	ELISA or POC assay	64.4 *	80.3 *	753	1072	Increased sensitivity when used in combination with cytology; allows separation of high vs. low-grade UC	[7,29]
CYFRA 21-1	Soluble fragments of cytoskeletal protein cytokeratin 19	Soluble	Protein	Immunoradio-metric assay or ELISA	82 *	80 *	1262	1233	High sensitivity for detection of high-grade and CIS tumors, poor sensitivity for early detection; generates false positive in inflammatory bladder conditions	[30] (Meta- analysis)
Apo-A1	Major high-density lipoprotein	Soluble	Protein	ELISA	89	85	223	153	Apolipoproteins are abundant in plasma; hence, urinary concentrations are affected by hematuria	[31]
					91.6	85.7	40	24		[32]
					95	92	86	62		[33]
IL-8	Leukocyte chemoattractant and angiogenic factor associated with inflammation and carcinogenesis	Soluble	Protein	ELISA	66.4 *	83.1 *	225	273	Urinary concentrations elevated in urothelial cell carcinoma	[34,35,36,37]
VEGF	Tumor angiogenesis factor	Soluble	Protein	ELISA	71.4 *	78.1 *	509	389	Secreted in urine by UC cells	[8,34,35,38,39,40]
CCL18	Cytokine involved in immunoregulatory and inflammatory processes; promotes cancer cells invasiveness	Soluble	Protein	ELISA	88	86	64	63	55 high-grade, 9 low-grade	[41]
Hyaluronidase/hyaluronic acid	Glycosidase that mainly degrades hyaluronic acid/glycosaminoglycan known to promote tumor metastasis and help avoid immune surveillance	Soluble	Protein	ELISA-like assay/zymography	90.8 *	82.5 *	981 participants		May permit early detection; high sensitivity and specificity for detection of both primary and recurrent tumors; further validation in larger, multi-center trials are required	[42] (meta- analysis)
sFAS	Anti-apoptotic protein released by UC cells	Soluble	Protein	ELISA	88	89.1	117	74	Better sensitivity in detecting low-grade UC than cytology	[43]
miRNA markers	Short non-coding RNAs that regulate gene expression by acting at the post-transcriptional level	Soluble or cellular	RNA (miRNA)	RT-PCR/NGS	75 *	75 *	719	494	Multi-miRNA assays have higher diagnostic sensitivity than single miRNA assays	[44] (meta- analysis)
					72 *	76 *	1556	1347		[45] (meta- analysis)
CD44/CD44 isoforms	Ubiquitously expressed transmembrane glycoprotein involved in cell–cell interactions, cell adhesion and migration	Soluble	RNA (mRNA)	RT-PCR	63.1	88.9	136	20	111 histological diagnosed UC, 25 benign urological disorders	[46]
		Cellular	Protein	ELISA	81	100	65	53	Presence of hematuria can interfere with the assay	[47]

Apo-A1, apolipoprotein-A1; BLCA-4, bladder cancer 4; C: control; CTCs, circulating tumor cells; hTERT, human telomerase reverse transcriptase; MCM, minichromosome maintenance; P, patient; POC, point-of-care; NGS, next-generation sequencing; sFas, soluble Fas; SN, sensitivity; SP, specificity; UBC, urinary bladder carcinoma antigen * Weighted or pooled sensitivity/specificity. ^ In vitro diagnostic test.

## Data Availability

No new data were created or analyzed in this study. Data sharing is not applicable to this article.
